# Supplementary material for: Association between oral microbiome and seven types of cancers in East Asian population: a two-sample Mendelian randomization analysis
Source: Front Mol Biosci. 2023 Nov 21;10:1327893. doi: 10.3389/fmolb.2023.1327893 (PMC10702768; doi:10.3389/fmolb.2023.1327893)
Supplement: Supplementary file 1 [file DataSheet1.ZIP › Supplementary Materials/MR plots for tongue/tongue═╝/Pancreatic cancer/╥╚╧┘░⌐_╔╕╤í╜ß╣√/pheno.1818_to_pancreatic cancer_leave_one_out.pdf]

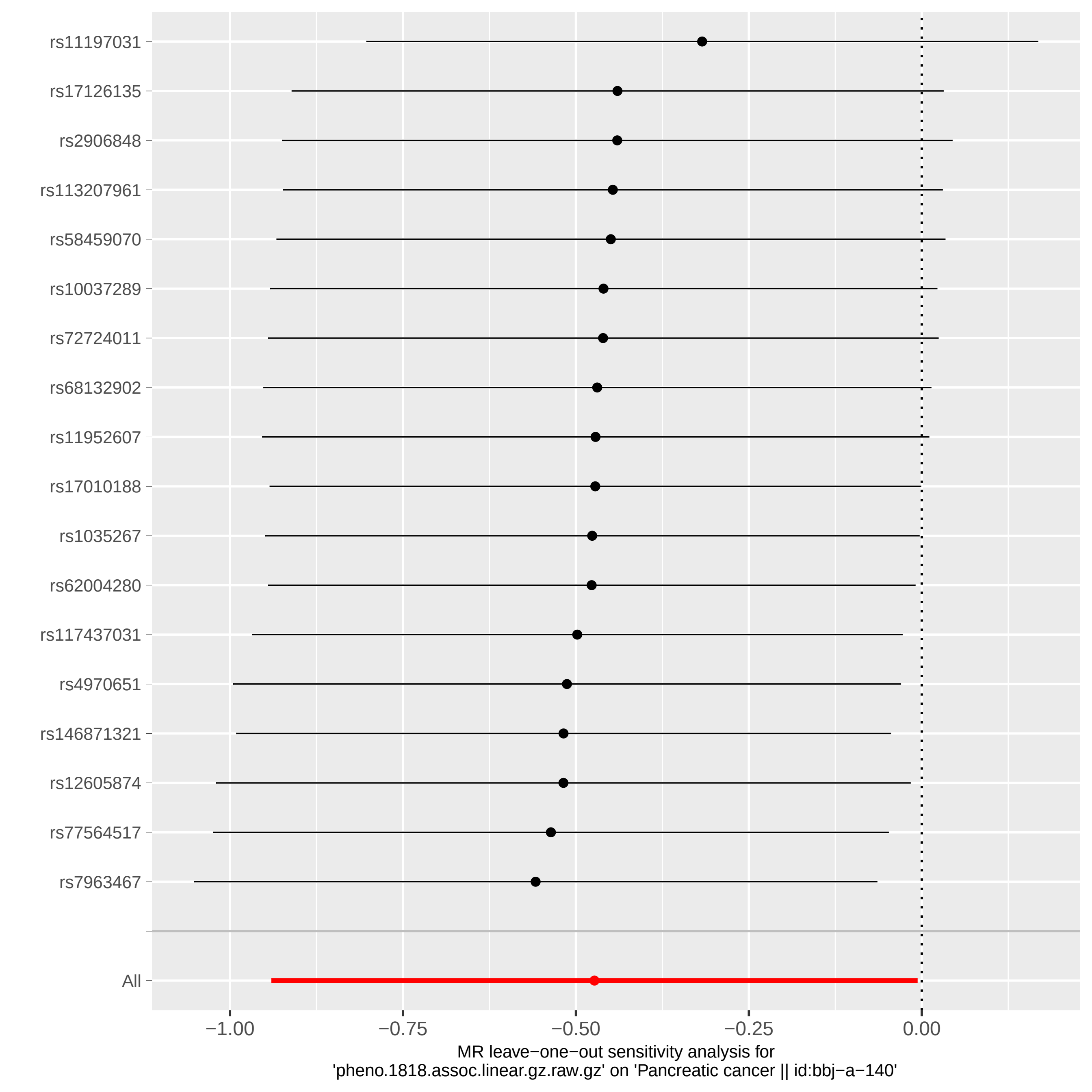
rs11197031
rs17126135
rs2906848
rs113207961
rs58459070
rs10037289
rs72724011
rs68132902
rs11952607
rs17010188
rs1035267
rs62004280
rs117437031
rs4970651
rs146871321
rs12605874
rs77564517
rs7963467
All
−1.00
−0.75
−0.50
−0.25
0.00
MR leave−one−out sensitivity analysis for
'pheno.1818.assoc.linear.gz.raw.gz' on 'Pancreatic cancer || id:bbj−a−140'
